# Rapid ovarian transcript changes during the onset of premature ovarian insufficiency in a mouse model

**DOI:** 10.1530/RAF-22-0036

**Published:** 2022-08-11

**Authors:** Heidy Kaune, Juan F Montiel, Mark Fenwick, Suzannah A Williams

**Affiliations:** 1 Laboratory of Reproduction, Centre for Biomedical Research, Faculty of Medicine, Universidad Diego Portales, Santiago, Chile; 2 Program of Ethics and Public Policies in Human Reproduction, Universidad Diego Portales, Santiago, Chile; 3 Laboratory of Integrative Neuroscience, Centre for Biomedical Research, Faculty of Medicine, Universidad Diego Portales, Santiago, Chile; 4 Academic Unit of Reproductive and Developmental Medicine, University of Sheffield, Sheffield, UK; 5 Nuffield Department of Women’s and Reproductive Health, Women’s Centre, John Radcliffe Hospital, University of Oxford, Oxford, UK

**Keywords:** ovary, fertility, oocyte

## Abstract

Premature ovarian insufficiency (POI) affects 1-3% of women under 40 years of age. The identified causes are highly heterogeneous, and 70% of the cases are idiopathic. The ovarian manifestation varies from a variable population of follicles that fail to develop (follicular POI) to the absence of follicles (afollicular POI) with a transition from one to the other over time. Previously, we have described a mouse model of POI that results from an oocyte-specific deletion of N- and O-glycans; Double Mutant (DM). DM females produce only one litter before undergoing POI due to ovarian dysfunction. In this study, we have characterised the gene expression profile of prepuberal (3 weeks), fertile (6 weeks) and infertile (9 weeks) DM ovaries. Up-regulation of cathepsin K (*Ctsk*, with unknown ovarian function) seems to trigger transcriptional changes in DM ovaries. Significant transcriptional changes then occur rapidly, associated with morphophysiological changes displayed by DM mice throughout the onset of POI. We identified genetic pathways such as extracellular matrix and immune response as candidates for the onset of POI in DM females. Remarkably, DM mice and POI women share a set of differentially expressed genes, including a functionally and co-expressed network of *Mcm* (minichromosome maintenance proteins) family members. The transcriptomic profile of the DM mouse model provides novel insight into the aetiology of POI.

**Lay summary:**

Problems in ovary function lead to reduced fertility or infertility. One such condition is premature ovarian insufficiency (POI) which affects 1% of women under 40 years of age, and in over 70% of these, the cause of POI is unknown. To investigate POI, we have developed a mouse model. These mice are initially fertile but develop POI by 3 months of age. In this study, we investigated the changes in genes activated in the ovaries during the transition from fertility to POI, and we did this by comparing them to normal mice; gene activation leads to molecule production. A molecule known as cathepsin K seems to trigger changes during the onset of POI, followed by others related to structure and immune response pathways. In addition, some genes were identified that are similar between the POI mice and POI women.

## Introduction

Premature ovarian insufficiency (POI, previously known as premature ovarian failure; POF) is a clinical condition characterised by hypergonadotropic hypogonadism and amenorrhoea that affects 1–3% of women under 40 years of age (Coulam *et al.* 1986, Meskhi & Seif 2006, Shelling 2010). The ovarian manifestation varies from a complete depletion of follicles (afollicular POI) to the presence of a variable population of follicles (follicular POI) that fail to develop (Meskhi & Seif 2006, Nelson 2009). The proportion of women with afollicular and follicular POI has been debated for several years between clinicians, and empirical studies have failed to provide reliable data. However, some studies indicate that the proportion of afollicular and follicular POI is fairly equal (Nelson *et al.* 1994), indicating that many women with follicular POI can potentially benefit from therapies focusing on reactivation of follicle development. Indeed, the analysis of ovarian cortical tissue has demonstrated that 54% of women with POI have residual follicles that can potentially be reactivated to resume fertility (Suzuki *et al.* 2015). However, in around 70% of the cases, POI is considered idiopathic (Meskhi & Seif 2006, Laissue *et al.* 2008, Rebar 2009, Vujovic 2009, Shelling 2010).

**Figure 1:**
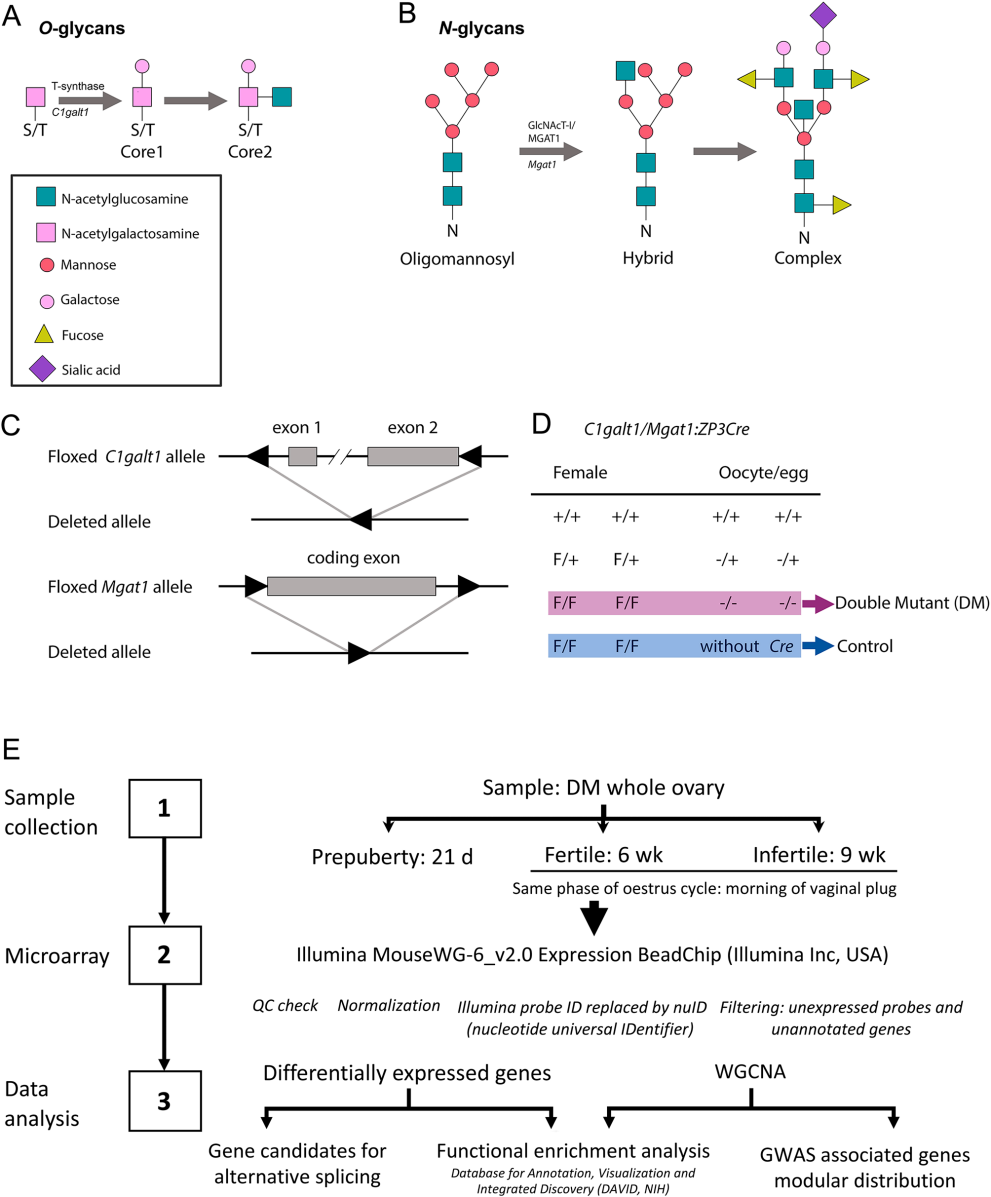
Mechanisms for generating complex *O*-glycans (A) and *N*-glycans (B). (C) Floxed and deleted *C1galt1* and *Mgat1* alleles. (D) Genotypes of female mice and the oocytes they generate after the action of the zona pellucida protein 3 a (ZP3) *Cre* transgene. F, floxed; +, WT; −, deleted gene. (E) Flowchart of microarray, functional enrichment, and weighted gene coexpression network analysis (WGCNA) (A, B, C, D; Modified from Williams & Stanley 2011 with permission).

Previously, we have described a mouse model of POI that results from the oocyte-specific deletion of two glycosyltransferases (Williams *et al.* 2007). These double mutant (DM) female mice have an oocyte-specific deletion of *Mgat1* and *C1galt1* genes, which encode for *N*-acetylglucosaminyltransferase 1 (GlcNAcT-1) and core 1 β1,3-galactosyltransferase (T-synthase) enzymes, respectively. These enzymes are required to generate hybrid *N*-glycans and complex *O*-glycans (core 1 and 2 *O*-glycans); therefore, any protein produced by oocytes from DM females will lack these glycans, including zona pellucida (ZP) proteins (Fig. 1A, B, C and D). Interestingly, DM females conditional knockout are fertile yet developed an altered ZP, indicating that these glycans are not required for fertilisation (Shi *et al.* 2004, Williams *et al.* 2007, Williams & Stanley 2009). DM females are fertile at 6 weeks of age, producing a smaller litter of around 50% of controls and then becoming unable to produce subsequent litters (Williams *et al.* 2007). With increasing age, ovarian morphology exhibits increasingly aberrant follicle development, and by 3 months of age, DM ovaries contain reduced numbers of follicles at later stages of follicular development. However, ovaries have an increased number of follicles at the primary stage, suggesting a block in follicle development (Williams *et al.* 2007). The ovarian failure observed in DM females by 3 months is consistent with the altered endocrine profile confirming the DM mouse as a model of POI (Williams & Stanley 2011).

The DM mouse has two main aspects that make it very attractive as a research model for POI. First, the DM phenotype is concordant with clinical features of hypogonadotropic hypogonadism observed in POI women. In this sense, the DM model represents an opportunity to study and explore possible mechanisms underlying the onset of POI that could potentially highlight candidate targets to re-establish follicle development.

Secondly, emphasising that the double deletion in the DM mouse is oocyte-specific, the model provides an opportunity to study the mechanisms through which the oocyte regulates ovarian physiology and, ultimately, fertility. The fact that DM females switch their phenotype from fertile to infertile in a narrow timeframe constitutes a remarkable opportunity to study the dynamics behind the regulation of fertility by the oocyte.

Several genes implicated in POI have been identified, including bone morphogenetic protein 15 (*BMP15*), fragile X mental retardation 1 (*FMR1*), and forkhead box L2 (*FOXL2*) (Cordts *et al.* 2011, Pouresmaeili & Fazeli 2014). Although these studies have contributed to establishing a list of potential candidate genes responsible for POI, understanding the mechanisms triggering the pathology based on the analysis of individual genes is very unlikely since the aetiology of POI is highly heterogeneous. A more comprehensive approach to identifying pathways and functions rather than identifying simply individual genes would benefit from unravelling the mechanisms underlying physiological or pathological processes. This would be accomplished by analysing global gene expression of all RNA molecules (including mRNA, rRNA, tRNA, and other ncRNA); this constitutes the transcriptome. Transcriptomes are specific to different organs and even individual cells. Transcriptomes can also be temporally regulated and changed during different functional states like different stages of the cell cycle or oestrous cycle, or exhibit circadian dynamics.

Hence, in this study, we have characterised the ovarian gene expression profile of prepubertal (3 weeks), fertile (6 weeks), and infertile (9 weeks) DM females, stages that reflect the onset of POI in these mice.

## Materials and methods

### Mice

Female mice carrying floxed *Mgat1* and *C1galt1* alleles and a ZP3*Cre* transgene were used as experimental females, whereas females carrying the floxed *Mgat1* and *C1galt1* alleles but not the ZP3*Cre* transgene were used as controls (Shi *et al.* 2004, Williams *et al.* 2007). Gene deletion by the ZP3*Cre* transgene was 100% efficient (Williams *et al.* 2007). Mice were kept under controlled conditions in 12 h light:12 h darkness cycles. All animal studies using mice were carried out with approval by the Local Ethical Review Committee (University of Oxford) under license, under the UK Animals (Scientific Procedures) Act 1986.

### Sample collection

Ovaries were collected from DM female mice at 3, 6, and 9 weeks of age. For 3-week samples, ovaries were dissected from females at exactly 21 days. To minimise variability in the ovaries collected at 6 and 9 weeks of age, samples were collected at the same oestrous cycle stage. To achieve this, females were housed with males and checked daily for the presence of vaginal plugs. Ovaries were collected at midday of the day a vaginal plug was found.

Dissection was carried out under sterile conditions, and ovaries were collected in a 1:1 solution of RNAlater® (Ambion, Applied Biosystems, Inc)/PBS. Ovaries were quickly and carefully cleaned of extra-ovarian tissue and bursa under a dissection microscope before freezing on dry ice and storage at −80°C.

### RNA extraction

Upon thawing, three ovaries from each age group were pooled into a single sample. Triplicate control and DM samples for each age (3, 6, and 9 weeks) were weighed, and RNA was extracted using a RiboPure kit (Ambion) according to the manufacturer’s instructions. Total RNA from each sample was quantified using a NanoDrop ND-1000 spectrophotometer (ThermoScientific), and the integrity of the RNA was assessed using a BioAnalyzer (Agilent 2100, Agilent Technologies). All samples were normalised to a concentration of 50 ng/µL, aliquoted, and stored at −80°C until analysis.

### Microarray analysis

A summarised workflow is presented in Fig. 1E. The whole-genome expression profile analysis was carried out using Illumina MouseWG-6_v2.0 Expression BeadChip (Illumina Inc), assessing 45,281 mouse transcripts per sample. Three chips were used to allocate the 18 samples analysed (triplicates of 3, 6, and 9 weeks control and DM samples). Hybridised samples were scanned using the Illumina BeadArray reader, and the results were extracted using Illumina GenomeStudio® data analysis software (Illumina, Inc). Expression profile results were analysed using RStudio (version 1.1.383, RStudio, Inc.) statistical software. RStudio platform was loaded with a series of packages required to analyse microarray data. Lumi (Du *et al.* 2008), a specific package for Illumina expression microarrays, was used for data input, quality control, variance stabilisation, normalisation, and gene annotation of probe expression profiles (Supplementary Figs 1 and 2, see section on supplementary materials given at the end of this article). Taking advantage of the technical replicates available on Illumina microarray, a variance-stabilising transformation (VST) algorithm (Lin *et al.* 2008) was applied to normalise raw data. Subsequent quantile normalisation was carried out to remove technical noise due to different hybridisation patterns between the Illumina chips. Annotated Illumina probe identifiers were replaced using nucleotide universal identifier (nuID, used as a source-independent naming convention for oligomers) to eliminate inconsistencies of identifiers between different chip versions (Du *et al.* 2007). Once non-annotated and unexpressed genes were filtered, differential expression analysis was carried out using linear models for microarray data in the limma package (Smyth 2004). Only genes with *P* values less than 0.05 using Benjamini–Hochberg (BH) correction were considered significant expression differences. Once analysed, gene official symbols and names were matched with the nuIDs of the differentially expressed transcripts.

### Functional enrichment analysis

The Database for Annotation, Visualisation, and Integrated Discovery (DAVID Bioinformatics Resources v6.8, National Institute of Allergy and Infectious Diseases, NIH, USA) was used to identify functionally enriched gene modules (Huang *et al.* 2009*a*, *b*). This database matches and clusters gene sets to establish their biological significance based on the Gene Ontology (GO) Consortium (http://geneontology.org). Differentially expressed genes in DM samples were divided into ‘upregulated’ and ‘downregulated’ from control, according to the fold-change value (Hong *et al.* 2014). The upregulated and downregulated list of genes were uploaded into DAVID environment and analysed by calling Functional Annotation Clustering. This DAVID environment reports clusters of similar annotations, allowing a more straightforward interpretation of the results and focusing on biological relevance. Genes highly associated with specific terms are clustered together, and the level of association is statistically measured by Fisher’s exact test. As an output, functional enrichment clusters, grouping several genes, were identified, and values of group enrichment score, the geometric mean of member’s *P* values (in negative log scale), and the Benjamini–Hochberg corrected *P*-value (*P*-value corrected for multiple hypothesis testing) were obtained. The top-ranked annotations groups based on the enrichment score most likely have lower *P* values for their annotation members.

### Identifying gene coexpression networks throughout WGCNA

We used weighted gene coexpression network analysis (WGCNA) developed by Horvath *et al.* (Zhang & Horvath 2005, Horvath & Dong 2008, Langfelder & Horvath 2008, Langfelder *et al.* 2011). A scale-free topology approximation was performed to identify the appropriate soft threshold (power); we used the lowest power (power = 6) that satisfied the approximate scale-free topology criterion where the topology _t index curve flattens out upon reaching a high value (in this case, roughly 0.6; Supplementary Fig. 3 and Supplementary Table 1) and produce manageable modules sizes. Those modules whose distance was less than 0.2 were merged, passing from 173 to 80 modules (Supplementary Tables 2 and 3). The resulting coexpression modules were related to functional annotations in DAVID environment as described before.

### Relating POI-genes with functional interaction networks

POI-associated genes were analysed using STRING 11 (http://string-db.org/), a database of known and predicted protein associations, which displays a network of nodes (genes) connected by edges representing functional relationships but not necessarily physical interactions (Supplementary Table 4).

### Graphic depictions

Graphic functional gene association and their representation in the coexpression networks were built using an edge-weighted spring embedding layout in Cytoscape 3.7.1 (Lopes *et al.* 2010), an open-source software platform for visualisation and annotation of networks. Edges across nodes/genes were visualised as a weighted thickness of human experimentally determined functional interactions from the STRING analysis. Gene coexpression module colour from the WGNA was assigned to the functional network nodes.

### Quantitative real-time polymerase chain reaction

To validate differential expression, cDNA was synthesised from 500 ng RNA per sample using random hexamer primers and SuperScript III reverse transcriptase (Invitrogen) in accordance with manufacturers’ instructions. cDNA samples were diluted two times in RNase-free water and stored at −20°C. For qPCR, a reaction mixture consisting of SYBR green and ROX (Kapa Biosystems Ltd, Potters Bar, UK), nuclease-free water, and 400 nM gene-specific primers for the following genes: *Ctsk, Igfbp2, Inhba, Bmp15, Gdf9, Bcl2l10, Foxo1,* and* Inha* were carried out along with the housekeeping gene *Atp5B* (ATP synthase, H+-transporting mitochondrial F1 complex, beta subunit). Each sample (1 µL) was assayed in duplicate on a 384-well plate alongside no-template (water) controls. An initial activation step at 95°C (3 min) preceded cycling for 40× at 95°C (3 s), 58°C (20 s), and 72°C (10 s) using an Applied Biosystems 7900HT Fast instrument (Applied Biosystems). Melt curves were included to ensure consistent and specific amplification of all products. *Atp5b* did not vary by more than one CT value across all samples and was therefore deemed an appropriate normaliser using the 2–−ΔΔCt method to calculate fold changes of each gene of interest (Livak & Schmittgen 2001).

## Results

### Differentially expressed genes/probes between control and DM samples

**Figure 2:**
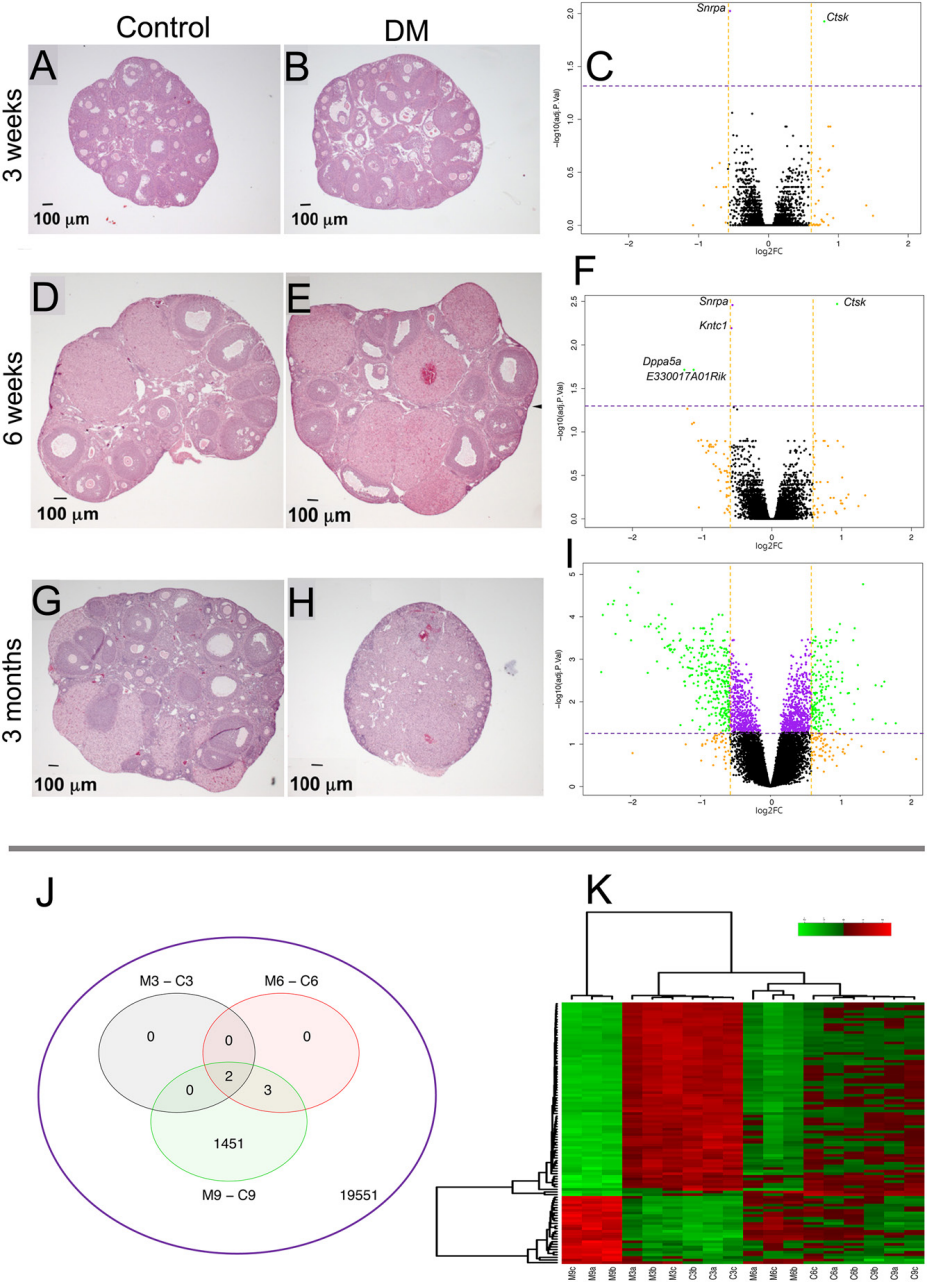
Evolution of gene expression changes throughout the onset of POI in DM ovaries. Morphological changes observed in control and DM ovaries from prepuberal to adult at 3 weeks (A and B), 6 weeks (D and E), and 3 months (G and H) are related to changes in gene expression represented in the volcano plots at 3 weeks (C), 6 weeks (F), and 9 weeks (I). All transcripts analysed are plotted according to the significance and fold-change (FC) on the Y- and X-axes, respectively. Purple dotted lines mark a significance level of 0.05. Orange dotted lines mark 1.5 FC compared to control samples. (J) Venn diagram representing all differentially expressed transcripts between control and DM samples at 3- (M3-C3), 6- (M6-C6), and 9-week (M9-C9). (K) Heatmap of top 100 differentially expressed genes between control and DM 9-week ovaries. Colours represent the level of gene expression per individual gene probe. The list of the gene probes analysed is at the right, and individual samples are at the bottom of the plot. The clustering of the samples and the gene probes is represented by the cladograms at the top and the left of the heatmap, respectively. Panels A, B, D, E, G, H, modified from Williams & Stanley 2011, with permission

Whole ovary RNA extraction was carried out in control and DM samples obtaining highly preserved RNAs. RNA integrity numbers (RIN) were as follows: control: RIN = 9.74 ± 0.06, DM RIN = 9.80 ± 0.11 (Supplementary Table 5). After normalisation and filtering of the data, a total number of 21,007 probes were analysed. At 3 weeks, there are minor morphological differences between control and DM samples, which are reflected by just two transcripts differentially expressed between conditions (Fig. 2A, B and C). We observed progressive, transcriptional and morphological changes, with five differentially expressed transcripts at 6 weeks (Fig. 2D, E and F). These differences increase dramatically by 9-week, when the number of probes differentially expressed is 1456, corresponding to 1253 genes (Fig. 2G, H, I and J). Interestingly, *Ctsk* was the only gene overexpressed in DM samples at 3 and 6 weeks among differentially expressed genes. Hierarchical clustering of the top 100 transcripts demonstrates that the expression profiles of DM and control samples at 6 and 9 weeks are distinctly different, while 3 weeks samples are indistinguishable (Fig. 2K).

The list of the differentially expressed genes in DM ovaries at 3- and 6 weeks and the top 10 genes at 9 weeks is presented in Table 1 (complete list in Supplementary material 1). At 3 weeks, DM ovaries differentially expressed small nuclear ribonucleoprotein polypeptide A (*Snrpa*), which is downregulated compared to control (log_2_FC= −0.55, Adj.*P* = 0.009) and *Ctsk* which is upregulated in DM samples (log_2_FC = 0.79, Adj.*P* = 0.01). At 6 weeks, three additional genes are also changed: Kinetochore associated 1 (*Kntc1*), developmental pluripotency associated 5A (*Dppa5a*), and RIKENcDNA E330017A01 gene (*E330017A01Rik*). Of the five genes differentially expressed at 6 weeks, only *Ctsk* is upregulated in DM samples; the other four are downregulated, being *Dppa5a* and *E330017A01Rik,* which displayed the lower expression from Control (log_2_FC = −1.25 and −1.12, respectively). At 9 weeks, 1456 genes/probes are differentially expressed in DM samples with *Ctsk* exhibiting persistent upregulation.

**Table 1:** Differentially expressed genes between DM and control mice.

Age/gene	Gene name	Log_2_FC	Adj. *P*-value
3 weeks			
*Snrpa*	Small nuclear ribonucleoprotein polypeptide A	−0.5508	0.0094
*Ctsk*	Cathepsin K	0.7989	0.0118
6 weeks			
*Ctsk*	Cathepsin K	0.9355	0.0033
*Snrpa*	Small nuclear ribonucleoprotein polypeptide A	−0.5646	0.0034
*Kntc1*	Kinetochore associated 1	−0.5790	0.0064
*Dppa5a*	Developmental pluripotency associated 5A	−1.2547	0.0193
*E330017A01Rik*	RIKEN cDNA E330017A01 gene	−1.12147	0.0193
9 weeks (top ten)			
*Apoa1*	Apolipoprotein A-I	−1.8921	8.63E-06
*Ctsk*	Cathepsin K	1.3272	1.71E-05
*Bcl2l10*	Bcl2-like 10	−2.0058	2.05E-05
*Apoa4*	Apolipoprotein A-IV	−1.8910	2.72E-05
*Gm15698*	Transcription elongation factor B (SIII) polypeptide 2 pseudogene	−2.2373	4.19E-05
*Nr5a2*	Nuclear receptor subfamily 5 group A member 2	−1.4089	5.05E-05
E330034G19Rik	RIKEN cDNA E330034G19 gene	−2.2523	5.05E-05
*Bmp15*	Bone morphogenetic protein 15	-2.3228	5.05E-05
*Gm15698*	Transcription elongation factor B (SIII) polypeptide 2 pseudogene	−2.0546	5.29E-05
*Kntc1*	Kinetochore associated 1	−07397	8.90E-05

### Functional enrichment analysis

The list of the 1253 differentially expressed genes detected in ovaries at 9 weeks was divided into upregulated and downregulated genes according to their positive or negative fold of change value (FC), respectively. Then, both sets of genes were uploaded and analysed separately in DAVID. Interestingly, the top five functional enrichment clusters for upregulated and downregulated genes are related to different cellular functions.

The top five functional enrichment clusters of up-regulated genes in 9-week DM ovaries are presented in Table 2. Interestingly, the first cluster is related to signal peptide and glycosylation, with 210 genes grouped in this cluster and a high enrichment score of 5.95. The following clusters are extracellular matrix, immune response, LPA (lipoprotein, propeptides, and anchored to the membrane), and cell adhesion.

**Table 2:** Top five functional enrichment clusters of upregulated and downregulated genes in 9 weeks DM ovaries.

Functional annotation	Genes, *n*	Enrichment score
Upregulated genes		
Signal peptide, glycosylation	210	5.95
Extracellular matrix	52	5.83
Immune response	61	3.26
LPA: lipoprotein; propeptide: removed in mature form; anchored to membrane	40	2.83
Cell adhesion	31	2.75
Downregulated genes		
Non-membrane-bounded organelle	134	22.04
Cell cycle	86	19.47
DNA replication	59	17.91
Chromosome structure	54	12.57
Nuclei and organelle lumen	82	10.53

The top five functional enrichment clusters for downregulated genes in 9-week DM ovaries are related to non-membrane bound organelles, cell cycle, DNA replication, chromosome structure, and nuclei and organelle lumen (Table 2). Compared to control, these clusters account for diminished cellular replication in 9-week DM ovaries.

### Follicular gene expression profile

**Figure 3:**
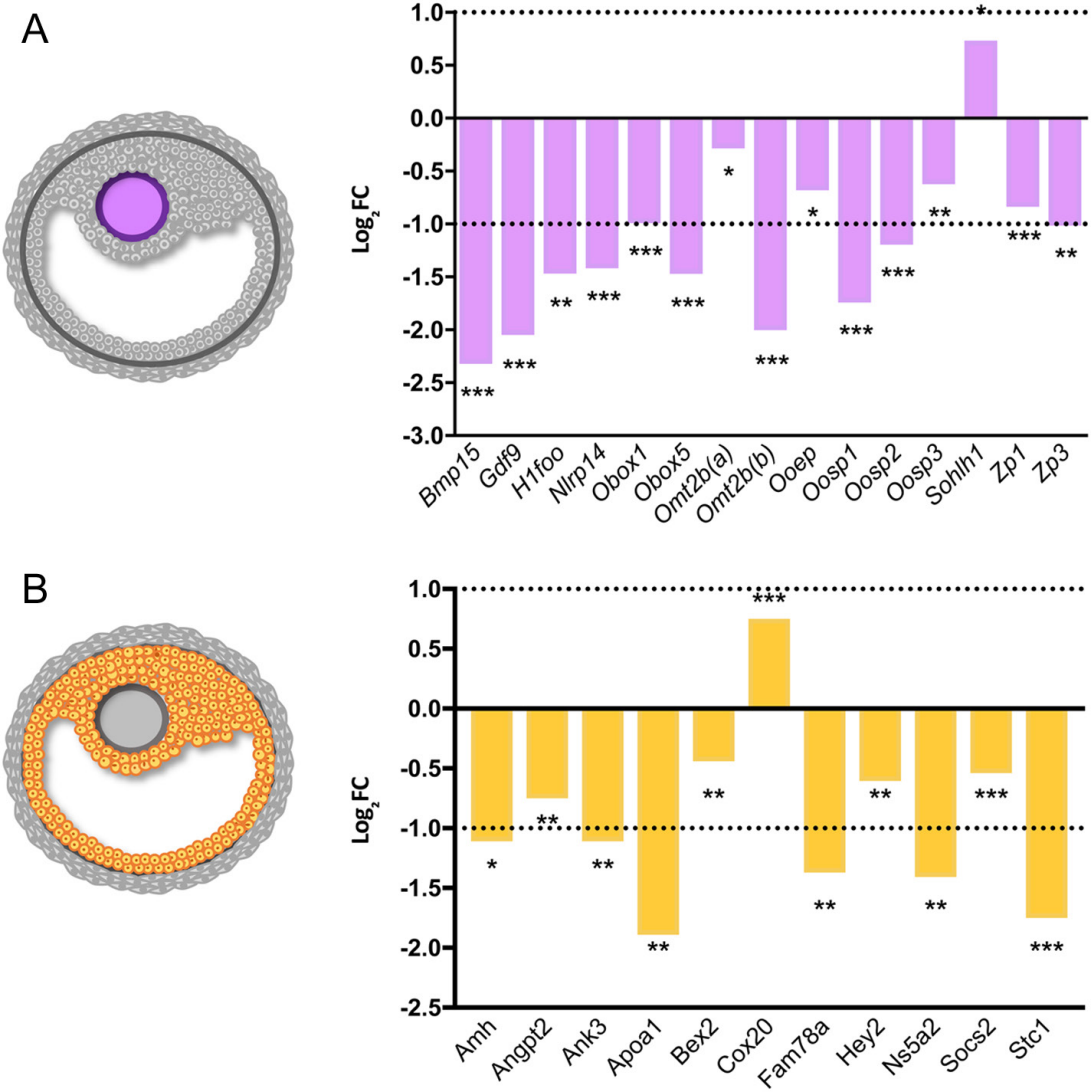
Expression profile of oocyte-secreted genes (A) and granulosa-secreted genes (B) in 9-week DM ovaries. Results are expressed as Log_2_FC of DM ovaries compared to control. Two probes tested Omt2b gene expression: *Omt2b(a)* and *Omt2b(b)*. Dotted lines indicate 2 and −2 FC from control. Asterisks represent adjusted *P*-value of gene expression compared to controls. ^*^P ≤ 0.05, ^**^P ≤ 0.01, ^***^P ≤ 0.001.

A subset of 15 oocyte-secreted genes and 11 granulosa cell-specific genes, extracted from consensus literature, was selected from differentially expressed genes at 9-week and plotted (Fig. 3). Theca cell-specific genes were not differentially expressed in our samples. In the oocyte subset of genes (Fig. 3A), we observed that only spermatogenesis- and oogenesis-specific basic helix-loop-helix 1 gene (*Sohlh1*) displayed overexpression in DM ovaries compared to control; the remaining 14 genes were downregulated in DM ovaries. A similar result was obtained from the granulosa cell compartment, with cytochrome c oxidase assembly protein 20 (*Cox20*) being the only transcript overexpressed from the 11 transcripts evaluated (Fig. 3B). DM ovaries at 9 weeks exhibit equivalent follicle count similar to controls; this finding could be explained by the blocked follicular growth observed at this stage (Williams & Stanley 2011). It is important to note that *Mgat1* and *C1galt1* expression levels were not expected to be, and were not, lower in the DM ovaries because the mutations are oocyte-specific, and the whole ovary transcriptome was analysed (Supplementary Table 6).

### Analysis of alternative splicing candidate genes

**Figure 4:**
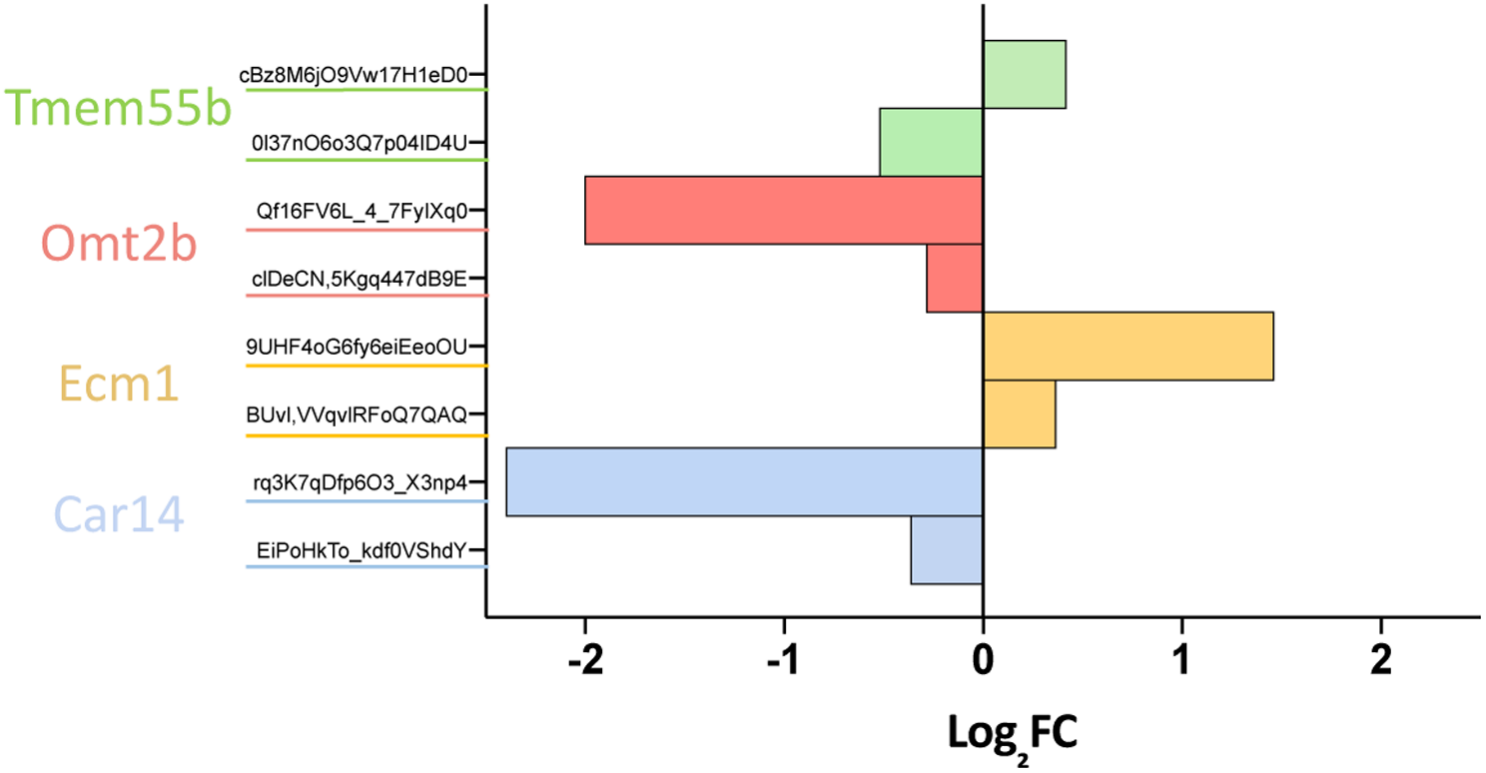
Splicing variant analysis of differentially expressed genes on 9-week DM samples. Bars of the same colours represent probes for the same gene. Results are expressed in log_2_FC of DM ovaries compared to control.

The Illumina chips used for this study have the advantage that to target some genes, multiple probes are used. Therefore, various regions of the gene are tested at the same time. This feature provides an opportunity to detect possible candidates for alternative splicing if the expression levels of the probes are discordant. Differentially expressed genes detected at 9-week targeted by more than one probe were tested. The genes with the most significant expression discrepancy between the probes were carbonic anhydrase 14 (*Car14*), extracellular matrix protein 1 (*Ecm1*), and oocyte maturation beta (*Omt2b*) (Fig. 4). The probes outlining these genes followed the same direction of expression; downregulation for *Car14* and *Omt2b* and upregulation for *Ecm1*. In addition, the two probes targeting the transmembrane protein 55B gene (*Tmem55b*) exhibited an opposite behaviour in terms of expression, one is downregulated (log_2_FC = −0.52), and the other one is upregulated (log_2_FC = 0.42), indicating *Tmem55b* as a candidate for alternative splicing in DM ovaries.

### WGCNA and modular functional enrichment analysis

**Figure 5:**
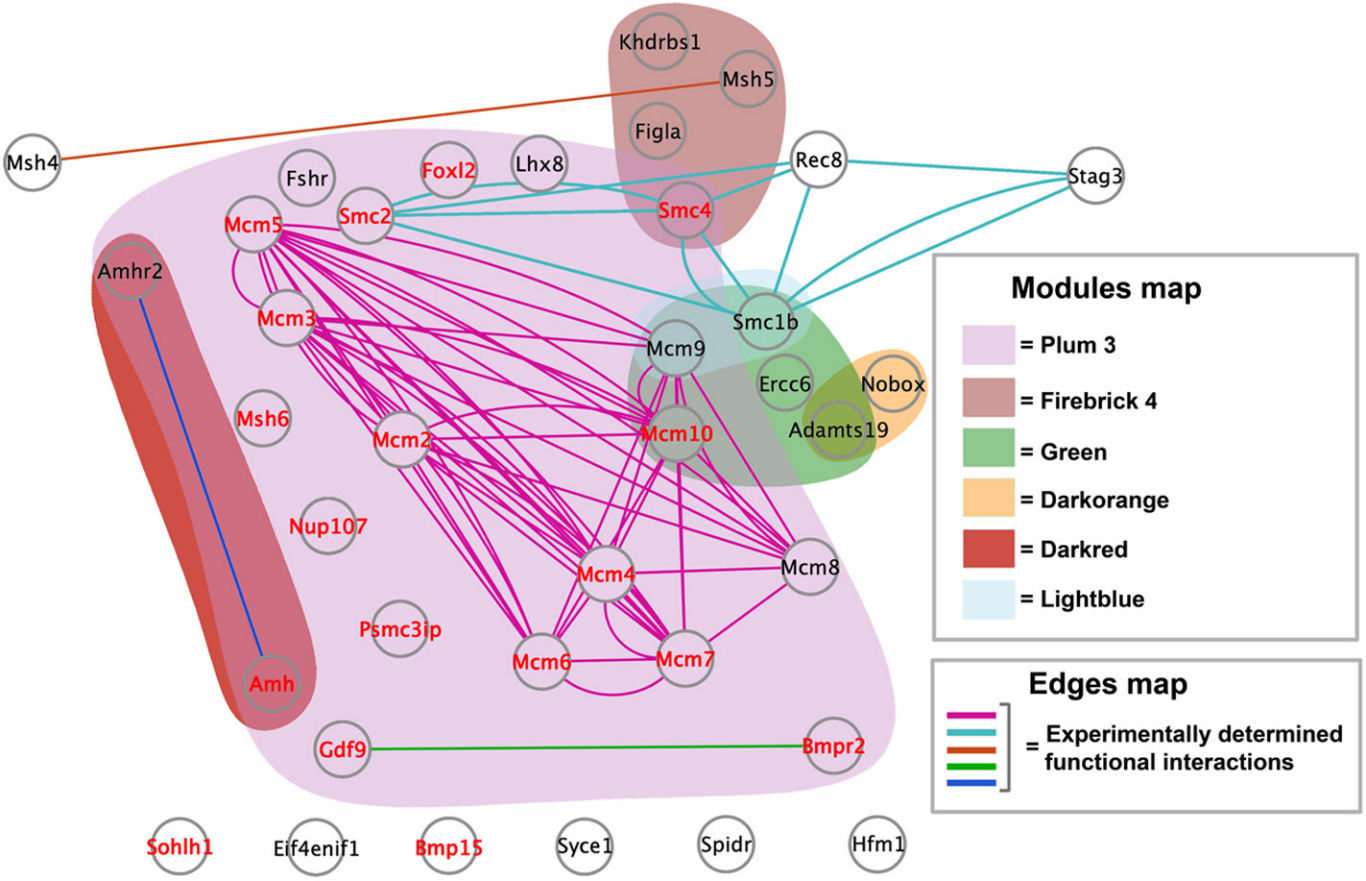
Functional network of 26 POI-associated genes in women, extracted from Jiao *et al.* (2018), plus 11 family-related genes differentially expressed in DM samples and their distribution in the corresponding WGCNA coexpression module. POI-associated genes previously described in women were differentially expressed in DM ovaries and distributed mainly in the Plum 3 module. STRING database analysis shows that these genes exhibit functional interactions, represented by coloured edges. Differentially expressed genes between control and DM samples are highlighted in red colour. For clarity, genes co-expressed with others than the POI-associated sub-list genes represented in this figure were not assigned to any module.

We obtained 80 coexpression modules containing between 31 and 4514 genes (Supplementary Fig. 4 and Supplementary Table 3). All modules exhibited significant functional enrichment in cellular processes and structures (Supplementary Material 2a and Supplementary Material 2b). Twenty-six currently identified causative and relevant candidate genes associated with POI phenotype (POI-genes) (Jiao *et al.* 2018) were analysed for their distribution in the coexpression modules and their potential functional interactions. Remarkably, 10 POI-associated genes (*AMHR2, BMPR2, FSHR, FOXL2, GDF9, LHX8, MCM8, MCM9, NUP107,* and *PSMC3IP*) were represented in the same module (Plum3, Fig. 5) with functional interactions with other genes across the same or other modules. POI genes in the Plum3 module participate in mitosis/meiosis and gene expression regulation. From the 26 POI genes analysed, a different set of seven mouse orthologue genes were differentially expressed in our DM samples (*Psmc3ip, Nup107, Sohlh1, Bmpr2, Foxl2, Gdf9, and Bmp15*). In addition, there are POI genes (*MSH4, MSH5, MCM8, MCM9, AMHR2, and SMC1b*) that are not differentially expressed in our DM samples. However, 11 additional genes that were related as family genes and/or members of functional interaction complexes with the genes above (*Msh6, Mcm2, Mcm3, Mcm4, Mcm5, Mcm6, Mcm7, Mcm10, Amh, Smc2,* and *Smc4*) were differentially expressed in the DM samples and included in this analysis. Sixteen out of the 18 POI-associated genes differentially expressed in the DM ovaries were co-expressed in the Plum3 module, the highest and most consistently correlated module across significantly different conditions (6 and 9 weeks; Supplementary Table 7).

## Discussion

Elucidating the genetic and molecular basis of POI remains a challenge in understanding ovarian physiology, causal mechanisms, and the heterogeneous aetiophysiopathology observed in women with POI (Jiao *et al.* 2018). Our analysis of the ovarian transcriptome of control and DM mice allowed us to detect differentially expressed genes, their functional enrichment, and gene networks that could support the similarities in the reproductive dynamics with POI in women.

Our differential gene expression analysis revealed that at 3 weeks, just two genes, small nuclear ribonucleoprotein polypeptide A (*Snrpa*) and cathepsin k (*Ctsk*), are differentially expressed in DM ovaries compared to controls. At 6-week, three more genes are differentially regulated: kinetochore associated 1 (*Kntc1*), developmental pluripotency associated 5A (*Dppa5a*), and RIKEN cDNA E330017A01 (*E330017A01Rik*). Interestingly, just 3 weeks later, at 9-week, differentially expressed genes in DM ovaries dramatically increased, reaching 1253 genes. This finding is consistent with the accelerated phenotype shift from fertile to infertile observed in DM ovaries (Grasa *et al.* 2012). At 3-week, prepuberal DM ovaries have displayed a normal response to superovulation with a comparable number of eggs ovulated in DM and control females; however, there were changes in ovarian morphology, including the presence of multiple-oocyte follicles (MOFs), ZP changes, and presence of granulosa cells beneath the ZP in some oocytes (Williams & Stanley 2011). Whether the downregulation of *Snrpa* and upregulation of *Ctsk* could be triggering DM ovarian phenotype at 3-week is still unclear.

Our transcriptomic analysis revealed that *Ctsk* is upregulated in DM ovaries in all stages analysed (3-, 6-, and 9-week). *Ctsk* and other seven differentially expressed genes were validated by RT-qPCR, demonstrating high correlations with microarray results at 3-, 6-, and 9-week stages (Supplementary Fig. 5). *Ctsk* encodes a cysteine protease expressed primarily in osteoclasts. Once secreted, CTSK degrades collagen and other matrix proteins during bone resorption; therefore, its role has been studied mainly in bone formation (Lotinun *et al.* 2013, Gentile *et al.* 2014, Jacome-Galarza *et al.* 2014). In this regard, CTSK is currently an important target for osteoporosis therapy (Cairoli *et al.* 2015). *Ctsk* mRNA expression changes during the oestrous cycle in mice, with maximum mRNA levels in the ovary from late pro-oestrus until meta-oestrus (Oksjoki *et al.* 2001). Immunohistochemistry revealed that CTSK was localised to the ovarian surface epithelium with levels highest in the contact points with secondary and Graafian follicles (Oksjoki *et al.* 2001). In calf oocytes, cathepsin family members (*CTSB, CTSK, CTSS*, and *CTSZ*) are expressed in higher levels at prepuberal rather than adult stages and significantly higher (all but *CTSK*) in cumulus cells of oocytes with reduced developmental competence (Bettegowda *et al.* 2008, Ashry *et al.* 2015). These results are concordant with our findings of lower developmental competence of DM oocytes, both *in vitro* and* in vivo*, observed during embryonic preimplantation development and follicle growth (Grasa *et al.* 2012, Kaune *et al.* 2016). In addition, CTSK dysregulation could explain the altered stroma rigidity that we reported previously in DM ovaries (Kaune *et al.* 2016). Although changes in protein expression levels do not correlate with global gene expression, differentially expressed genes are better correlated with protein expression than global gene expressions (Koussounadis *et al.* 2015). Nevertheless, further analyses are needed to establish a correlation across relevant transcripts and protein expression level changes in DM (e.g. *Snrpa* and *Ctsk*).

The functional enrichment analysis of the sharp increase of differentially expressed genes in 9-week DM ovaries was carried out independently for upregulated and downregulated genes. Interestingly, the top five annotation clusters of upregulated genes are related to glycosylation, extracellular matrix, immune response, propeptides, and cell adhesion (Table 2). Meanwhile, the top five annotation clusters for downregulated genes are related to non-membrane-bounded organelles, cell cycle, DNA replication, chromosome structure, and nuclei and organelle lumen (Table 2). Cell cycle-related function was downregulated, probably due to the marked reduction in ovarian function observed in 9-week DM females. On the other hand, and considering that DM females produce oocytes lacking complex and hybrid *N*-glycans and core 1-derived *O*-glycans and that glycans are essential regulators of cellular communication, the fact that this ‘stimuli’ is inducing an upregulation in cell signalling and glycosylation activity is both intriguing and fascinating to the understanding of the ovarian physiology in the DM model since it could be indicating a compensatory mechanism. This finding highlights the oocyte as a regulator of fertility and ovarian function and identifies post-translational processing as a new area that warrants investigation to understand infertility-related pathologies. Functional studies, for example, testing the effect of cathepsin K on follicle development *in vitro* using cathepsin K inhibitors to attempt recovering of follicle development would be valuable to get a comprehensive understanding of how the oocyte–somatic cell communication is taking place in the DM ovary and to contribute to our knowledge of the molecular mechanisms involved in fertility regulation by the oocyte.

Since the DM female mouse has an oocyte-specific deletion, a subset of 15 genes encoding oocyte-secreted proteins was extracted from the list of differentially expressed genes at 9-week and was analysed in the fold of change from control samples (Fig. 3A). DM females display an arrested follicle development, but the total number of follicles (and therefore oocytes) remains unchanged between DM and controls (Williams & Stanley 2011). Thus, gene expression changes of oocyte-secreted proteins are not related to a decreased number of oocytes in the DM ovary, although all stages of oocyte development are not represented. Fourteen genes were downregulated, of which 71.4% (10 genes) displayed an expression level below two-fold compared to control samples. From these, *Bmp15* and *Gdf9* exhibited a higher decrease in gene expression. Reduced expression of *BMP15* and *GDF9* has been reported in ovaries of women with polycystic ovary syndrome (PCOS) (Teixeira Filho *et al.* 2002, Zhao *et al.* 2010, Wei *et al.* 2011, 2014), and these studies agree that this is directly related with the aberrant follicle development and infertility observed in these women; consistent with the arrest of follicle development and infertility observed in 9-week DM ovaries. Of the 15 oocyte-expressed genes analysed, *Sohlh1* is the only upregulated gene in DM ovaries. *Sohlh1* in mouse ovaries showed that the protein is expressed preferentially in the oocyte. Its disruption in *Sohlh1* knockout mice leads to disruption in follicle development; therefore, it is critical for normal fertility (Pangas *et al.* 2006). Its function when upregulated is unknown. Targeting gene sequencing of *SOHLH1* in Han Chinese and Serbian women with non-syndromic POI revealed the presence of ten heterozygous variants that may suggest a role of *SOHLH1* in POI (Zhao *et al.* 2015). Moreover, exome sequencing analysis performed in two pairs of sisters with non-syndromic hypergonadotropic hypogonadism from two unrelated families revealed the presence of mutations in *SOHLH1* (Bayram *et al.* 2015). These studies targeted this gene as a candidate for ovarian dysfunction for the first time in humans.

In addition to the analysis of the oocyte-specific gene, 11 granulosa cell-specific genes extracted from the differentially expressed genes at 9 weeks were analysed independently (Fig. 3B). Interestingly, only *Cox20* was upregulated in DM samples, and even though its expression has been documented in ovary (Fagerberg *et al.* 2014), its role in ovarian function has not been reported. One of the major granulosa-specific genes downregulated in DM ovaries was *Apoa1*. This gene encodes apolipoprotein A-I, which is the main protein component of high-density lipoprotein in plasma, and it has been related to hormonal dysregulation in PCOS (Zheng *et al.* 2017, Zhang *et al.* 2018). Another gene highly downregulated was *Stc1*, which has been implicated in multiple reproductive functions such as a marker for implantation in pigs (Song *et al.* 2009), a modulator of redox status in swine granulosa cells (Baioni *et al.* 2011), and marker to choose the best embryo to transfer and predict pregnancy outcome in both human (Wathlet *et al.* 2012) and cows (Mazzoni *et al.* 2017). As expected, *Amh* was also downregulated in DM ovaries since this gene has been recognised as a reproductive health marker for fertility decline, including POI (Jankowska 2017).

A meta-analysis carried out on data extracted from 59 publications (5333 patients and 9399 controls) found an association between five gene (*BMP15*, *ESR1*, *FMR1*, *FSHR,* and *INHA*) polymorphisms and the risk of POI (Pu *et al.* 2014). From these five genes, bone morphogenetic protein 15 (*Bmp15*) and inhibin alpha (*Inha*) were differentially expressed in DM ovaries, being both genes downregulated in 9-week DM ovaries compared to controls (log_2_FC = −2.32, Adj.*P* = 5.05e-05 and log_2_FC = −1.02, Adj.*P* = 0.0007, respectively). *Bmp15* encodes a protein that is a member of the transforming growth factor-beta superfamily, and along with *Gdf9,* it is involved in oocyte maturation and follicular development (Yan *et al.* 2001, Su *et al.* 2004, Fenwick *et al.* 2013, Peng *et al.* 2013). Defects in *Bmp15* have been related to ovarian pathologies and infertility, including altered ovulation (McIntosh *et al.* 2012), PCOS (Wei *et al.* 2014), and POI (Pu *et al.* 2014). *Inha* encodes the alpha subunit of inhibin A and inhibin B protein complexes. Inhibin A is implicated in granulosa cell proliferation and differentiation in the ovary, androgen synthesis, and oocyte development (Knight & Glister 2006) and constitutes a candidate gene for POI (Chand *et al.* 2010).

Our microarray gene expression analysis enables us to explore potential gene candidates for alternative splicing in those genes exhibiting discordant expression patterns among probes. This analysis revealed that only three genes showed considerable differences in the expression levels among probes: *Car14*, *Ecm1,* and *Omt2b*. These changes followed the same direction of change; upregulation for the two probes targeting *Car14* and *Omt2b* genes and downregulation for the two probes targeting *Ecm1*. Interestingly, a fourth gene, transmembrane protein 55B (*Tmem55b*), exhibited the opposite direction of change for its two gene probes.

The different nature and functional enrichment of differentially expressed genes among control and DM ovaries enlightened us to explore a gene network involvement rather than a gene-specific causative mechanism involved in the onset of POI in our mouse model. WGCNA uses a 'scale-free' network assumption that is a good approximation to biological gene coexpression networks, which can be compared across individuals, samples, and cell types. WGCNA is based upon gene coexpression correlations, and the resulting coexpression modules can be related to relevant ovarian physiological processes like cell cycle control, gene regulation, and metabolism. We observed modularity in the gene pattern expression (Supplementary Fig. 6) with significant functional enrichment in cellular processes and structures that matched the human POI genes' functional description. We took advantage of an available dataset of 26 identified causative and relevant candidate genes associated with POI phenotype (Jiao *et al.* 2018) to interrogate them in the resulting WGCNA modules. The presence of an important number of POI genes and others closely related to them in only one network module (Plum3) supports the notion of a biological gene pathway involved in the DM phenotype that is partially shared by described POI cases in women (Thanatsis *et al.* 2019, Yatsenko & Rajkovic 2019, Veitia 2020, Wang *et al.* 2020).

Furthermore, available functional association and protein–protein interaction databases allowed us to analyse if these networks of genes can jointly contribute to a shared function, but this does not necessarily mean they are physically binding to each other (Szklarczyk *et al.* 2017). For example, our analysis can detect transcriptional activity controlled by cis-regulatory elements of non-coding DNA neighbouring specific genes in addition to protein–protein interactions. Our analysis displayed a network of POI genes connected by edges representing functional relationships experimentally validated in the main coexpression module as the Plum3 one. We consider this evidence of a complex functional genomic architecture underlying POI, assembled by biological pathways involving multiple nodes (genes) representing different entrances or causative targets to the POI phenotype. For instance, *Smc1b*, *Amhr2,* and *Smc4* genes co-expressing but participating in different functional interaction networks (Fig. 5).

In summary, transcriptomic analyses of DM ovaries demonstrated a sharp change in gene expression compared to controls from 6 weeks, when females are fertile, to 9 weeks, when DM females are infertile. Remarkably, differentially expressed genes at 9 weeks in the DM ovaries are mainly functionally enriched for cell communication and extracellular matrix, representing potential pathways affecting ovarian function and impairing fertility capacity. These candidate genes and pathways should be explored in depth to understand how the oocyte triggers these events. Undoubtedly, these results will provide a solid foundation for future studies to contribute to our understanding of how the oocyte orchestrates ovarian physiology and fertility. The results of the coexpression and functional network analysis demonstrated multiple potential pathways associated with POI phenotype in our mouse model, and further functional analysis would enable us to understand and identify the complexity of pathways interactions underlying POI aetiology, allowing us to improve risk prediction, prompt diagnosis and earlier intervention and treatment for POI women.

## Supplementary Material

Supplementary Material

Supplementary Figure 1

Supplementary Figure 2

Supplementary Figure 3

Supplementary Figure 4

Supplementary Figure 5

Supplementary Figure 6

Supplementary Data 1

Supplementary Data 2

Supplementary Data 3

## Declaration of interest

Suzannah A Williams is an Editor of Reproduction and Fertility. Suzannah A Williams was not involved in the review or editorial process for this paper, on which she is listed as an author. The other authors declare no conflict of interest.

## Funding

This work was supported by a grant awarded from the Medical Research Council to S W (Grant number G0900058). H K was a recipient of Becas Chile PhD scholarship from the Government of Chile and PhD scholarship for Academics from Diego Portales University, Chile.

## Data availability

The microarray data will be available on Gene expression Omnibus (GEO).

## Author contribution statement

H K and S W contributed to the conception and study design. H K conducted the experiments. M F conducted validation experiments. H K, S W, and J F M analysed and interpreted the data. H K, S W, J F M, and M F wrote and revised the manuscript.
