# Supplementary material for: Rapid ovarian transcript changes during the onset of premature ovarian insufficiency in a mouse model
Source: Reprod Fertil. 2022 Aug 11;3(3):173–86. doi: 10.1530/RAF-22-0036 (PMC9513667; doi:10.1530/RAF-22-0036)
Supplement: Supplementary Material [file supplementary_material.pdf]

SUPPLEMENTARY TABLE 1: WGCNA statistics

	Power	SFT.R.sq	slope	truncated.R.sq	mean.k.	median.k.	max.k.
1	1	0.22600	1.820	0.847	6550.0	6650.00	9020
2	2	0.00614	-0.144	0.800	2970.0	2960.00	5230
3	3	0.26000	-0.854	0.828	1600.0	1540.00	3470
4	4	0.46000	-1.210	0.858	961.0	882.00	2470
5	5	0.55900	-1.440	0.873	619.0	539.00	1850
6	6	0.60600	-1.590	0.883	420.0	345.00	1440
7	7	0.65200	-1.670	0.903	297.0	231.00	1150
8	8	0.68000	-1.740	0.914	216.0	159.00	931
9	9	0.68400	-1.830	0.911	162.0	113.00	769
10	10	0.70600	-1.870	0.921	124.0	81.60	644
11	12	0.73900	-1.890	0.935	76.7	45.20	466
12	14	0.75000	-1.930	0.936	50.2	26.60	349
13	16	0.77000	-1.910	0.942	34.3	16.50	269
14	18	0.83200	-1.830	0.975	24.2	10.70	211
15	20	0.85100	-1.850	0.984	17.6	7.11	173

SUPPLEMENTARY TABLE 2: Original module colors and gene numbers per module

aliceblue	antiquewhite1	antiquewhite2	antiquewhite4
46	57	71	97
bisque4	black	blue	blue1
111	511	837	40
blue2	blue3	blue4	blueviolet
82	52	64	64
brown	brown1	brown2	brown3
768	51	83	39
brown4	chocolate3	chocolate4	coral
112	46	57	72
coral1	coral2	coral3	coral4
99	97	71	56
cornflowerblue	cyan	darkgoldenrod4	darkgreen
46	238	41	169
darkgrey	darkmagenta	darkolivegreen	darkolivegreen1
160	142	143	52
darkolivegreen2	darkolivegreen4	darkorange	darkorange2
64	83	157	115
darkred	darkseagreen1	darkseagreen2	darkseagreen3
173	47	57	72
darkseagreen4	darkslateblue	darkturquoise	darkviolet
100	110	165	81
deeppink	deeppink1	deeppink2	firebrick
64	51	39	41
firebrick2	firebrick3	firebrick4	floralwhite
52	66	83	123
green	green3	green4	greenyellow
644	47	59	289
grey60	honeydew	honeydew1	indianred1
202	73	100	41
indianred2	indianred3	indianred4	ivory
52	66	84	123
lavender	lavenderblush	lavenderblush1	lavenderblush2
31	47	59	73
lavenderblush3	lightblue2	lightblue3	lightblue4
100	42	52	67
lightcoral	lightcyan	lightcyan1	lightgreen
85	204	124	196
lightpink	lightpink1	lightpink2	lightpink3
34	47	59	73
lightpink4	lightskyblue3	lightskyblue4	lightslateblue
101	42	53	67
lightsteelblue	lightsteelblue1	lightyellow	magenta
85	124	190	494
magenta1	magenta2	magenta3	magenta4
34	49	60	73
maroon	mediumorchid	mediumorchid4	mediumpurple
102	96	43	53
mediumpurple1	mediumpurple2	mediumpurple3	mediumpurple4
67	86	124	71
midnightblue	mistyrose	mistyrose4	moccasin
221	55	35	50
navajowhite	navajowhite1	navajowhite2	navajowhite3
61	74	102	46
orange	orange4	orangered	orangered1
158	43	53	68
orangered3	orangered4	paleturquoise	paleturquoise4
87	132	144	36
palevioletred	palevioletred1	palevioletred2	palevioletred3
50	61	75	104
pink	pink2	pink3	pink4
506	43	53	68
plum	plum1	plum2	plum3
87	133	107	81
plum4	powderblue	purple	purple2

64	51	289	38
red	royalblue	royalblue2	royalblue3
641	176	38	51
saddlebrown	salmon	salmon1	salmon2
148	254	63	76
salmon4	sienna1	sienna2	sienna3
105	44	53	142
sienna4	skyblue	skyblue1	skyblue2
69	150	88	94
skyblue3	skyblue4	slateblue	slateblue1
135	71	54	45
steelblue	tan	tan2	tan3
144	265	38	51
tan4	thistle	thistle1	thistle2
63	79	105	106
thistle3	thistle4	tomat	tomato2
81	63	51	38
turquoise	violet	white	whitesmoke
1448	143	156	45
yellow	yellow2	yellow3	yellow4
705	54	69	91
yellowgreen			
136			

SUPPLEMENTARY TABLE 3: Merged module colors and gene numbers per module

aliceblue	antiquewhite1	blue1	blue3
46	57	40	52
blue4	blueviolet	brown1	brown2
742	661	447	1088
brown3	brown4	chocolate3	chocolate4
99	4514	249	57
coral	cornflowerblue	darkgoldenrod4	darkgreen
56	46	41	169
darkolivegreen1	darkolivegreen2	darkolivegreen4	darkorange
52	64	83	157
darkred	darkseagreen1	darkseagreen2	darkseagreen3
173	47	57	333
darkseagreen4	darkslateblue	firebrick2	firebrick4
100	228	52	706
floralwhite	green	green3	green4
123	1988	47	59
honeydew	indianred1	indianred2	indianred4
73	84	52	184
ivory	lavender	lavenderblush	lavenderblush2
617	31	97	73
lightblue2	lightblue3	lightcoral	lightcyan1
42	181	363	124
lightpink	lightpink3	lightpink4	lightskyblue3
34	73	101	111
lightskyblue4	lightsteelblue	magenta1	magenta2
53	393	34	49
magenta4	mediumorchid	mediumorchid4	mediumpurple
73	96	43	53
mediumpurple2	mediumpurple4	mistyrose	mistyrose4
86	71	55	35
moccasin	navajowhite1	navajowhite3	paleturquoise4
50	74	46	265
palevioletred1	palevioletred2	pink2	plum3
61	242	43	3356
royalblue	royalblue2	royalblue3	salmon1
247	38	51	108
salmon4	sienna4	tan2	tan3
105	69	38	51
thistle2	tomato	tomato2	yellow2
106	51	38	54

SUPPLEMENTARY TABLE 4: String interactions of POF-associated genes

#node1	node2	node1_string_internal_id	node2_string_internal_id	node1_external_id	node2_external_id	homology	experimentally_determined_interaction
SMC4	SMC2	1852454	1847012	9606.ENSP00000341382	9606.ENSP00000286398	0.624	0.997
MCM2	MCM6	1845676	1845487	9606.ENSP00000265056	9606.ENSP00000264156	0.773	0.992
MCM3	MCM5	1843226	1842548	9606.ENSP00000229854	9606.ENSP00000216122	0.761	0.99
MCM7	MCM5	1848945	1842548	9606.ENSP00000307288	9606.ENSP00000216122	0.786	0.989
MCM7	MCM4	1848945	1845065	9606.ENSP00000307288	9606.ENSP00000262105	0.802	0.989
MCM7	MCM3	1848945	1843226	9606.ENSP00000307288	9606.ENSP00000229854	0.797	0.989
MCM4	MCM5	1845065	1842548	9606.ENSP00000262105	9606.ENSP00000216122	0.803	0.989
MCM6	MCM5	1845487	1842548	9606.ENSP00000264156	9606.ENSP00000216122	0.771	0.989
MCM2	MCM3	1845676	1843226	9606.ENSP00000265056	9606.ENSP00000229854	0.759	0.989
MCM2	MCM5	1845676	1842548	9606.ENSP00000265056	9606.ENSP00000216122	0.798	0.989
MCM7	MCM6	1848945	1845487	9606.ENSP00000307288	9606.ENSP00000264156	0.804	0.989
MCM6	MCM4	1845487	1845065	9606.ENSP00000264156	9606.ENSP00000262105	0.758	0.989
MCM2	MCM4	1845676	1845065	9606.ENSP00000265056	9606.ENSP00000262105	0.772	0.989
MCM7	MCM2	1848945	1845676	9606.ENSP00000307288	9606.ENSP00000265056	0.795	0.989
MCM4	MCM3	1845065	1843226	9606.ENSP00000262105	9606.ENSP00000229854	0.747	0.988
MCM6	MCM3	1845487	1843226	9606.ENSP00000264156	9606.ENSP00000229854	0.756	0.97
MCM10	MCM2	1854261	1845676	9606.ENSP00000354945	9606.ENSP00000265056	0	0.934
MCM9	MCM5	1849712	1842548	9606.ENSP00000314505	9606.ENSP00000216122	0.714	0.929
MCM9	MCM4	1849712	1845065	9606.ENSP00000314505	9606.ENSP00000262105	0.702	0.929
MCM9	MCM3	1849712	1843226	9606.ENSP00000314505	9606.ENSP00000229854	0.692	0.929
MCM8	MCM4	1856948	1845065	9606.ENSP00000368174	9606.ENSP00000262105	0.741	0.917
MCM8	MCM7	1856948	1848945	9606.ENSP00000368174	9606.ENSP00000307288	0.748	0.917
MCM9	MCM7	1849712	1848945	9606.ENSP00000314505	9606.ENSP00000307288	0.754	0.916
MCM8	MCM5	1856948	1842548	9606.ENSP00000368174	9606.ENSP00000216122	0.729	0.883
MCM8	MCM9	1856948	1849712	9606.ENSP00000368174	9606.ENSP00000314505	0.718	0.872
MCM10	MCM6	1854261	1845487	9606.ENSP00000354945	9606.ENSP00000264156	0	0.861
MCM10	MCM4	1854261	1845065	9606.ENSP00000354945	9606.ENSP00000262105	0	0.83
MCM10	MCM3	1854261	1843226	9606.ENSP00000354945	9606.ENSP00000229854	0	0.82
MCM9	MCM6	1849712	1845487	9606.ENSP00000314505	9606.ENSP00000264156	0.703	0.819
MCM8	MCM2	1856948	1845676	9606.ENSP00000368174	9606.ENSP00000265056	0.744	0.819
MCM10	MCM7	1854261	1848945	9606.ENSP00000354945	9606.ENSP00000307288	0	0.807
SMC1B	REC8	1853538	1849097	9606.ENSP00000350036	9606.ENSP00000308699	0	0.782
MCM10	MCM9	1854261	1849712	9606.ENSP00000354945	9606.ENSP00000314505	0	0.755
MCM8	MCM3	1856948	1843226	9606.ENSP00000368174	9606.ENSP00000229854	0.699	0.72
SMC4	REC8	1852454	1849097	9606.ENSP00000341382	9606.ENSP00000308699	0	0.679
STAG3	REC8	1850137	1849097	9606.ENSP00000319318	9606.ENSP00000308699	0	0.676
SMC1B	SMC4	1853538	1852454	9606.ENSP00000350036	9606.ENSP00000341382	0.628	0.669
MCM10	MCM5	1854261	1842548	9606.ENSP00000354945	9606.ENSP00000216122	0	0.578
AMHR2	AMH	1844545	1842844	9606.ENSP00000257863	9606.ENSP00000221496	0	0.558

BMPR2	GDF9	1856145	1847804	9606.ENSP00000363708	9606.ENSP00000296875	0	0.558
MCM8	MCM1 0	1856948	1854261	9606.ENSP00000368174	9606.ENSP00000354945	0	0.528
SMC1B	STAG3	1853538	1850137	9606.ENSP00000350036	9606.ENSP00000319318	0	0.512
MSH5	MSH4	1856362	1845301	9606.ENSP00000364855	9606.ENSP00000263187	0.639	0.412

SUPPLEMENTARY TABLE 5: Quantity and integrity of RNA obtained per sample

**Sample**			**RNA concentration (ng/μl)**	**28S/18S**	**A260/A280**	**A260/A230**	**RIN**
**Control**	3W	A	429.4	1.4	2.04	2.05	9.8
		B	181.5	1.1	2.06	2.11	10
		C	293.4	1.1	2.06	2.1	10
	6W	A	464.9	1.4	2.04	2.16	9.8
		B	401.7	1.1	2.04	2.12	9.6
		C	470	1.3	2.05	2.18	9.8
	9W	A	567.6	1.1	2.09	1.95	9.5
		B	861	1.5	2.12	2.21	9.7
		C	408.8	1.3	2.04	2.16	9.5
**DM**	3W	A	216.3	1.2	2.08	2.19	10
		B	105.7	1.4	2.04	1.95	10
		C	200.5	1.5	2.12	2.17	10
	6W	A	432.5	1.8	2.08	2.16	9
		B	456.8	1.4	2.06	1.95	9.9
		C	571.2	1.7	2.13	2.17	9.9
	9W	A	580.2	1.9	2.14	2.13	9.8
		B	408.8	1.5	2.08	2.17	9.8
		C	575.9	1.5	2.12	2.16	9.5

SUPPLEMENTARY TABLE 6: Mutated genes did not display significant whole ovary gene expression differences between mutant and control conditions across any analysed stages. More experiments are needed at single egg levels as mutations are oocyte specific.

Age	geneSymbol	geneName	logFC	AveExpr	t	P.Value	adj.P.Val	B
3 weeks	Mgat1	mannoside acetylglucosaminyltransferase 1	-0.0995256	8.86130784	-1.481123	0.15979322	0.99914962	-4.7376602
	Mgat1	mannoside acetylglucosaminyltransferase 1	0.00405141	8.6870717	0.05938482	0.95344937	0.99914962	-5.7009994
	C1galt1	core 1 synthase, glycoprotein-N-acetylgalactosamine 3-beta-galactosyltransferase, 1	0.08408748	8.88630502	1.284344	0.21899227	0.99914962	-4.9633439
6 weeks	Mgat1	mannoside acetylglucosaminyltransferase 1	0.01232988	8.86130784	0.18349119	0.85693207	0.99990036	-5.8525449
	Mgat1	mannoside acetylglucosaminyltransferase 1	0.0995793	8.6870717	1.45961654	0.16553853	0.90969796	-4.895197
	C1galt1	core 1 synthase, glycoprotein-N-acetylgalactosamine 3-beta-galactosyltransferase, 1	0.01433655	8.88630502	0.21897515	0.82969564	0.99990036	-5.8455341
9 weeks	Mgat1	mannoside acetylglucosaminyltransferase 1	-0.0998139	8.86130784	-1.4854133	0.15866724	0.46241991	-5.4990736
	Mgat1	mannoside acetylglucosaminyltransferase 1	0.00947633	8.6870717	0.13890242	0.89142167	0.96797948	-6.5642904
	C1galt1	core 1 synthase, glycoprotein-N-acetylgalactosamine 3-beta-galactosyltransferase, 1	-0.0442756	8.88630502	-0.6762613	0.50942782	0.78722599	-6.338499

SUPPLEMENTARY TABLE 7: As a result of the WGCNA the eigengene clustering, the Plum3 module exhibits the highest and more consistent correlations across significantly different conditions (6 and 9 weeks) samples. The module eigengene is the first principal component representing the Plum3 gene expression profile.

**Phenotype**	**Sample name**	**MEplum3**
Control	C6a	0.181539
	C6b	0.196006
	C6c	0.335885
	C9a	0.208619
	C9b	0.253596
	C9c	0.387849
Double mutant	M6a	-0.01306
	M6b	-0.12641
	M6c	-0.20629
	M9a	-0.46065
	M9b	-0.38379
	M9c	-0.3733
