# Supplementary figures and images for: Rapid ovarian transcript changes during the onset of premature ovarian insufficiency in a mouse model
Source: Reprod Fertil. 2022 Aug 11;3(3):173–86. doi: 10.1530/RAF-22-0036 (PMC9513667; doi:10.1530/RAF-22-0036)

**A**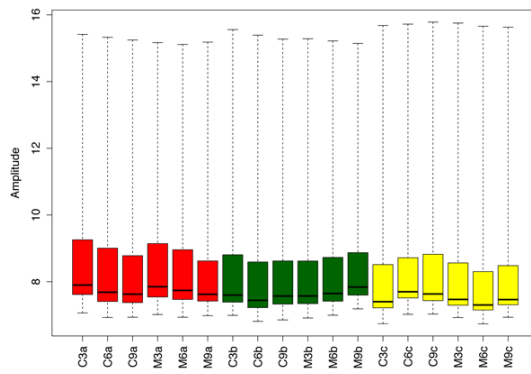**B**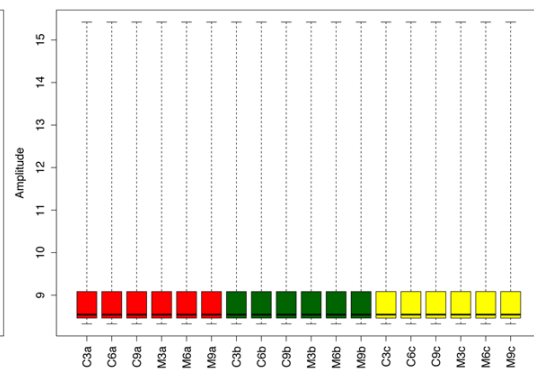**C**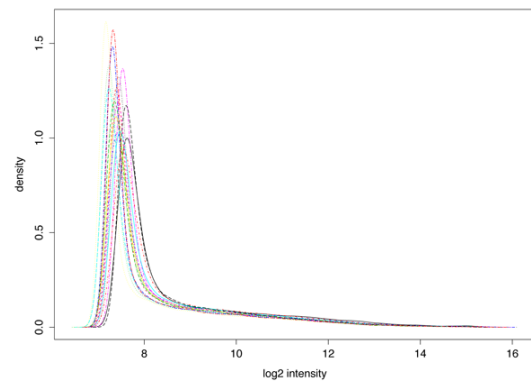**D**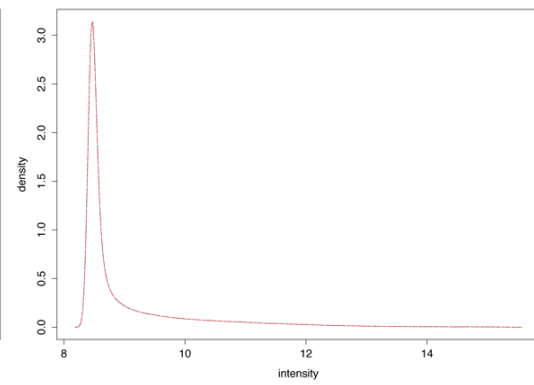

Supplementary Figure 1

Supplement: Supplementary Figure 1 [file supplementary_figure_1.pdf]

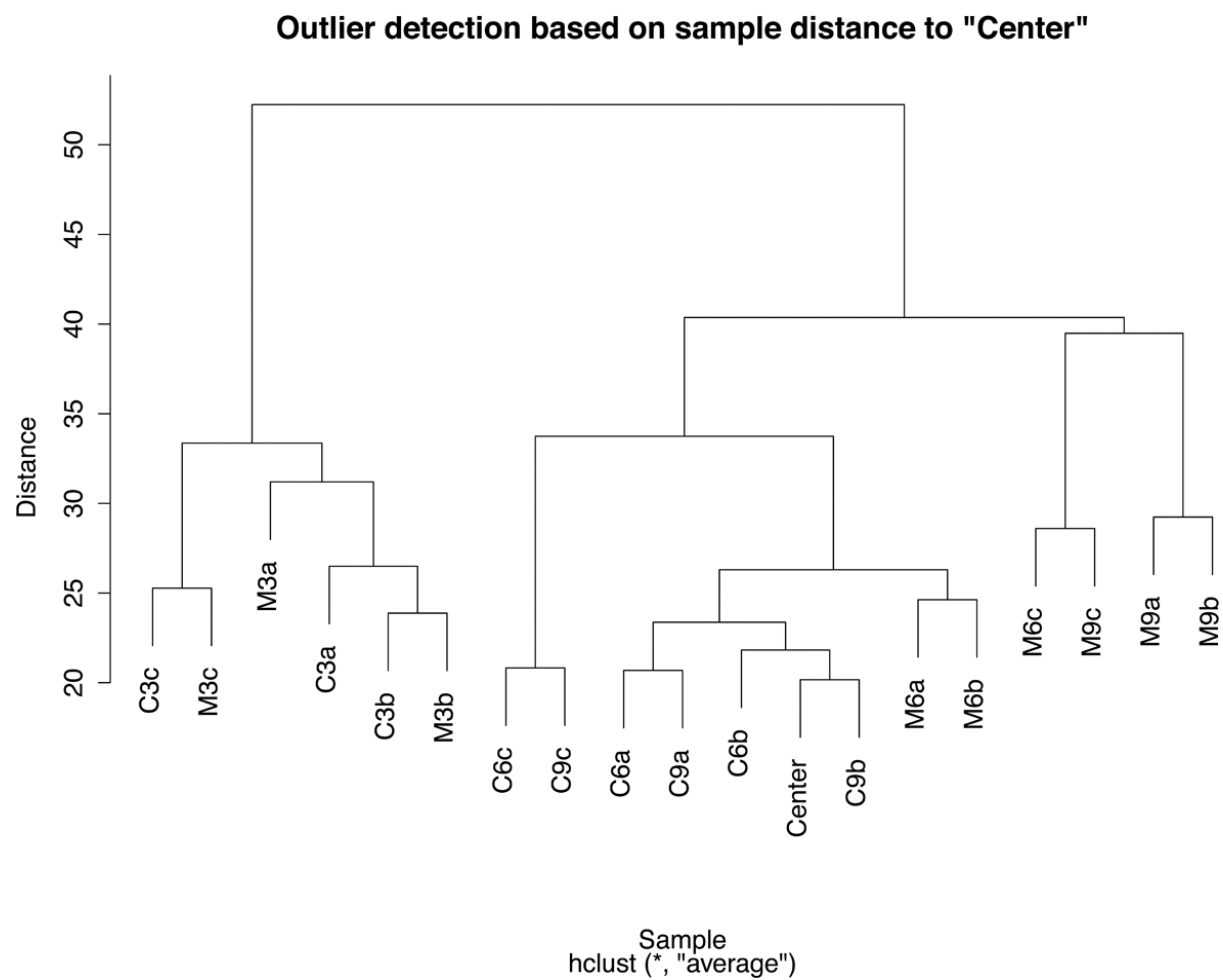

Supplementary Figure 2

Supplement: Supplementary Figure 2 [file supplementary_figure_2.pdf]

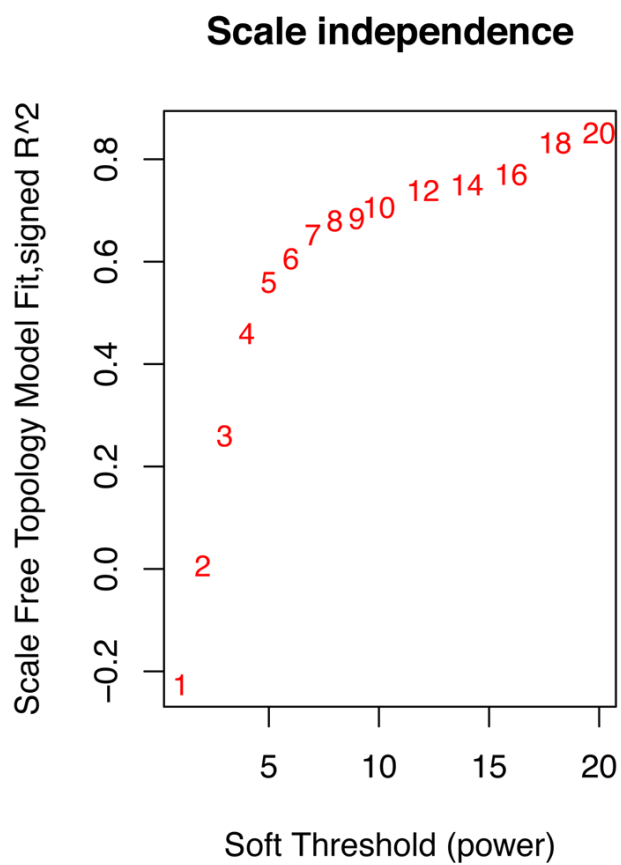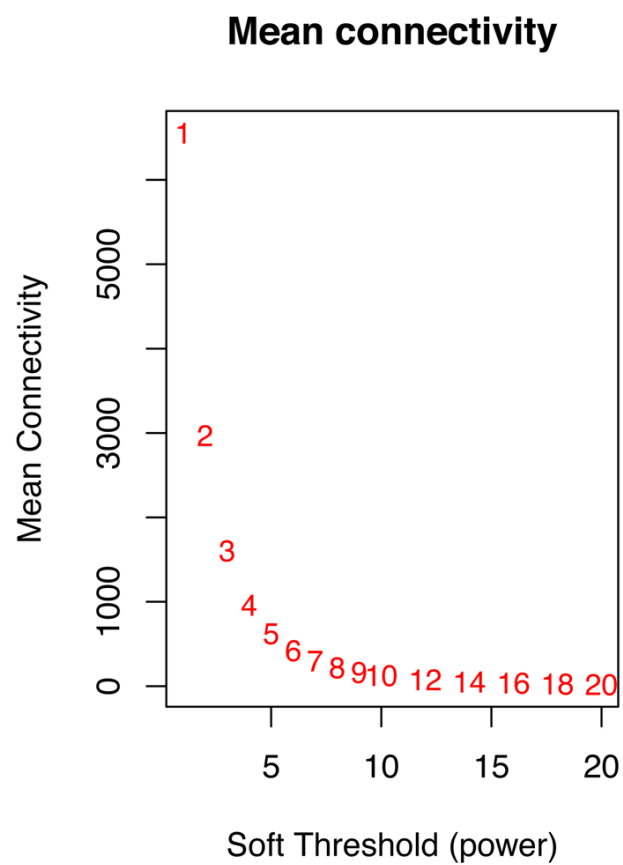

Supplementary Figure 3

Supplement: Supplementary Figure 3 [file supplementary_figure_3.pdf]

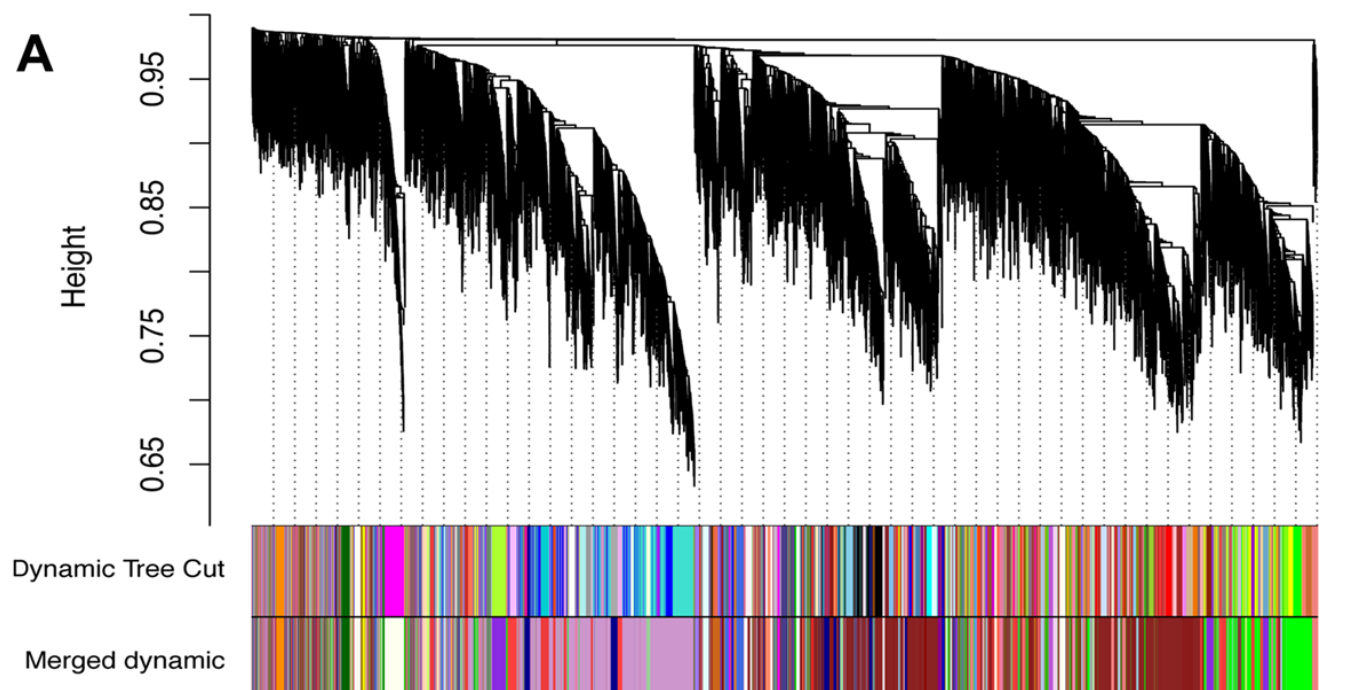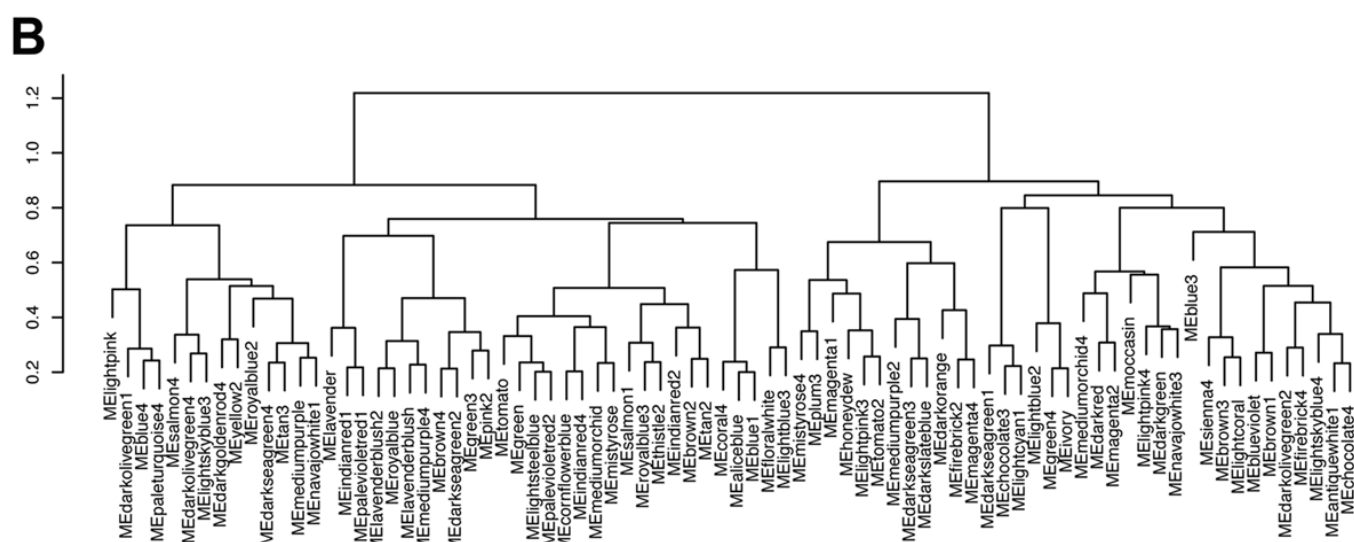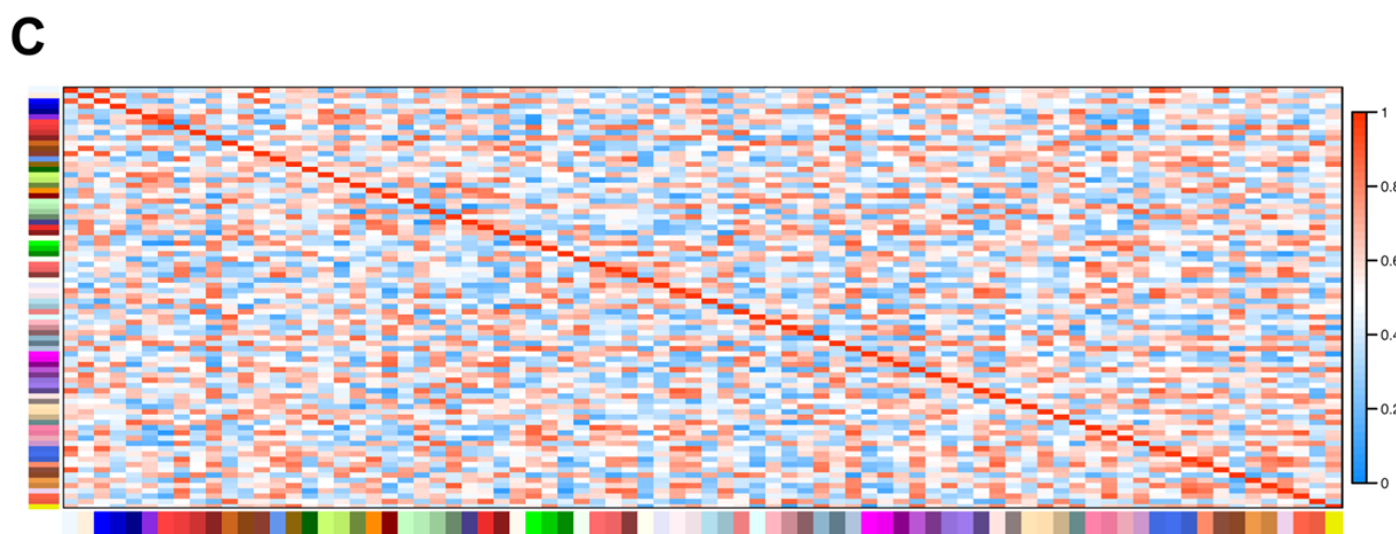

Supplementary Figure 4

Supplement: Supplementary Figure 4 [file supplementary_figure_4.pdf]

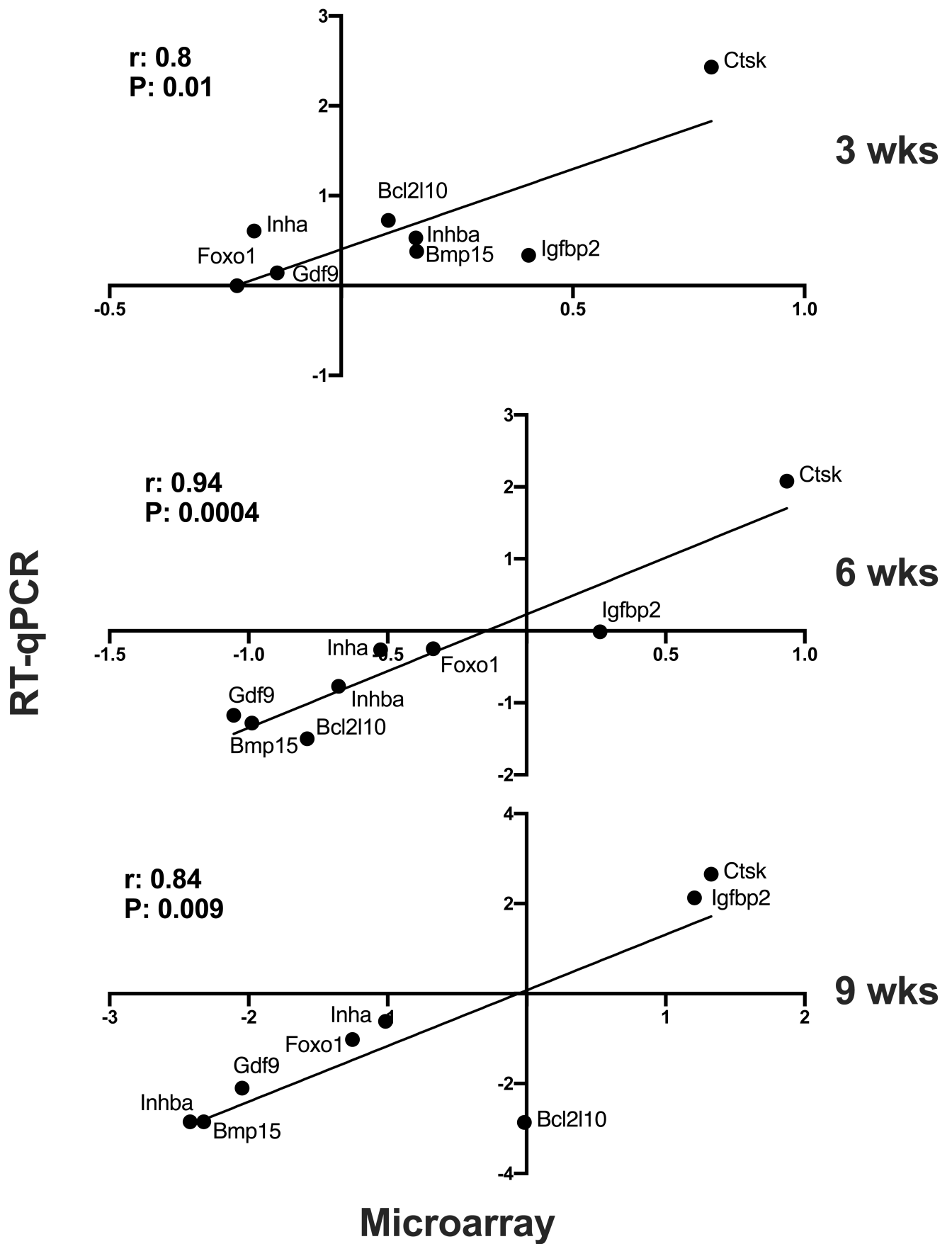

Supplementary Figure 5

Supplement: Supplementary Figure 5 [file supplementary_figure_5.pdf]

### Network heatmap plot, selected genes

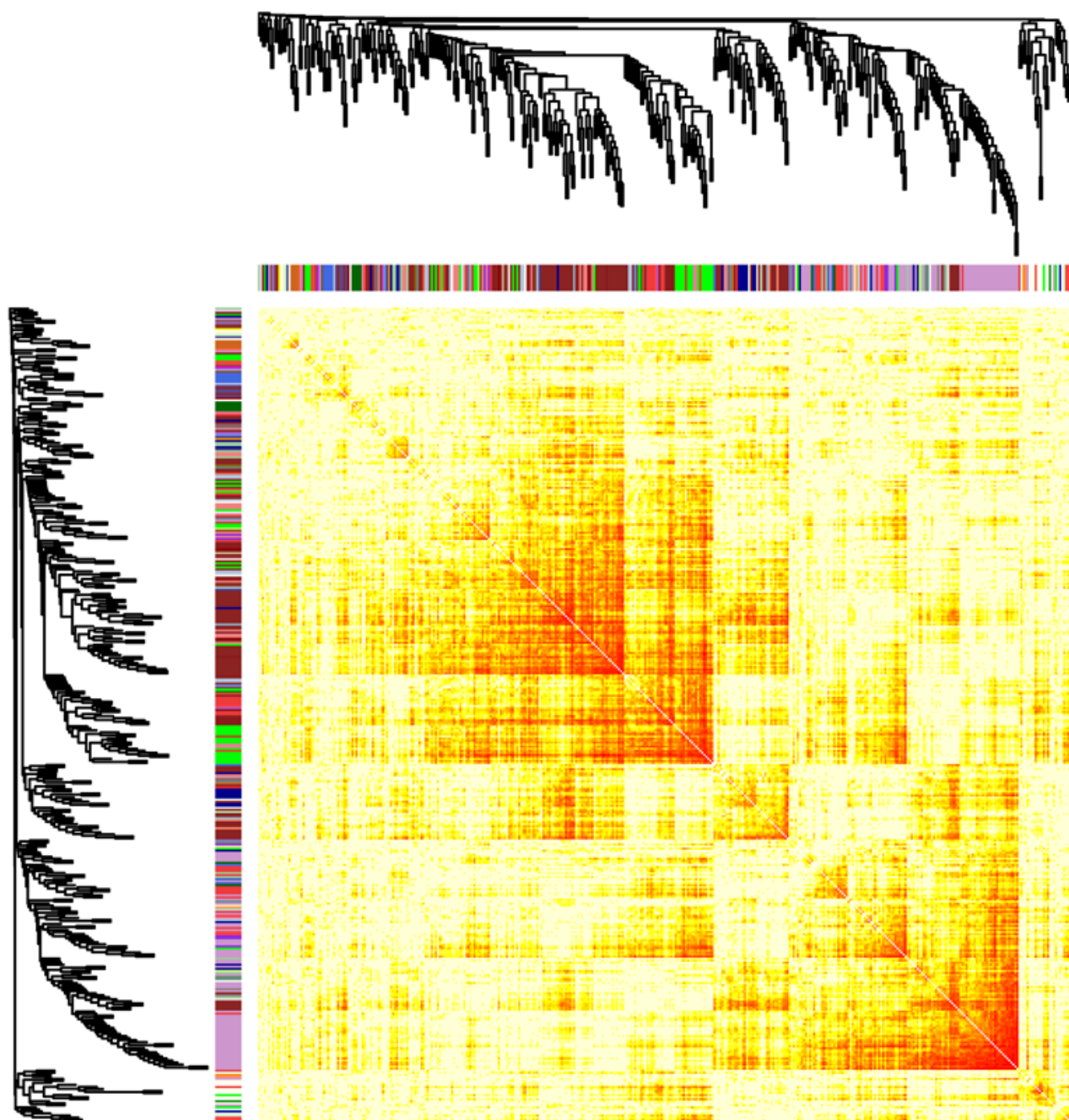

Supplementary Figure 6

Supplement: Supplementary Figure 6 [file supplementary_figure_6.pdf]
